# Addressing emerging issues in entomology: 2023 student debates

**DOI:** 10.1093/jisesa/ieae080

**Published:** 2024-08-02

**Authors:** Victoria Pickens, Jacqueline Maille, William Jacob Pitt, Jordan Twombly Ellis, Sara Salgado, Kelly M Tims, Carla-Cristina Edwards, Malcolm Peavy, Zia Valerie Williamson, Tyler R T Musgrove, Ethan Doherty, Arjun Khadka, Allyson Martin Ewert, Tanner C Sparks, Bandana Shrestha, Hazel Scribner, Navi Balthazor, Rachel L Johnson, Chip Markwardt, Rupinder Singh, Natalie Constancio, Kayleigh C Hauri, John J Ternest, Scott W Gula, DeShae Dillard

**Affiliations:** Department of Entomology, Kansas State University, Manhattan, KS, USA; Department of Entomology, Kansas State University, Manhattan, KS, USA; Tree Fruit Research & Extension Center, Washington State University, Wenatchee, WA, USA; Department of Entomology, Texas A&M University, College Station, TX, USA; Department of Entomology and Nematology, University of Florida, Fort Pierce, FL, USA; Department of Entomology, University of Georgia, Athens, GA, USA; Department of Entomology and Nematology, University of California-Davis, Davis, CA, USA; Department of Entomology, University of Georgia, Athens, GA, USA; Department of Entomology, University of Georgia, Athens, GA, USA; Department of Entomology, Louisiana State University, Baton Rouge, LA, USA; Department of Mathematical and Statistical Sciences, Clemson University, Clemson, SC, USA; Department of Forestry and Environmental Sciences, Clemson University, Clemson, SC, USA; Department of Entomology, Louisiana State University, Baton Rouge, LA, USA; Department of Entomology, Louisiana State University, Baton Rouge, LA, USA; Department of Entomology, Louisiana State University, Baton Rouge, LA, USA; Department of Entomology, Louisiana State University, Baton Rouge, LA, USA; Department of Entomology, Kansas State University, Manhattan, KS, USA; Department of Entomology, Kansas State University, Manhattan, KS, USA; Department of Entomology, Kansas State University, Manhattan, KS, USA; Department of Entomology, Kansas State University, Manhattan, KS, USA; Department of Entomology, Kansas State University, Manhattan, KS, USA; Department of Entomology, Michigan State University, East Lansing, MI, USA; Department of Entomology, Michigan State University, East Lansing, MI, USA; Department of Entomology and Nematology, University of Florida, Gainesville, FL, USA; Department of Forestry and Natural Resources, Purdue University, West Lafayette, IN, USA; Department of Entomology, Michigan State University, East Lansing, MI, USA

**Keywords:** artificial intelligence, science communication, honey bee, native pollinator, food security

## Abstract

The Entomological Society of America (ESA) Student Debates is an annual student competition at the ESA Annual Meeting organized by Student Debates Subcommittee (SDS) members of the ESA Student Affairs Committee. In conjunction with the 2023 ESA Annual Meeting theme, ‘Insects and influence: Advancing entomology’s impact on people and policy’, the theme of this year’s student debate was ‘Addressing emerging issues in entomology’. With the aid of ESA membership, the SDS selected the following debate topics: (1) Should disclosure of artificial intelligence large language models in scientific writing always be required? and (2) Is it more important to prioritize honey bee or native pollinator health for long-term food security within North America? Four student teams from across the nation, composed of 3–5 student members and a professional advisor, were assigned a topic and stance. Over the course of 5 months, all team members researched and prepared for their assigned topic before debating live with an opposing team at the 2023 ESA Annual Meeting in National Harbor, Maryland. SDS members additionally prepared and presented introductions for each debate topic to provide unbiased backgrounds to the judges and audience for context in assessing teams’ arguments. The result was an engaging discussion between our teams, judges, and audience members on emerging issues facing entomology and its impact on people and policy, such as scientific communication and food security, that brought attention to the complexities involved when debating topics concerning insects and influence.

## Introduction

Each year, students across the nation come together to compete in the Entomological Society of America (ESA) Student Debates at the ESA Annual Meeting. Organized by the Student Debates Subcommittee (SDS) of the ESA Student Affairs Committee (SAC), this annual event showcases students’ abilities to research, develop, and present persuasive arguments for popular entomological topics based on evidence and logic, as well as rapidly evaluate and counter-argue opposing stances in front of a live audience. By organizing the student debates, ESA and the SAC hope to provide students the opportunity to practice and demonstrate their professional skills, as well as foster intellectual engagement and constructive dialogue on popular complex topics in the entomological community.

As outlined by Parker et al. (2019), the debate theme and topics generated and selected by the SDS address present-day topics and issues relevant to the ESA membership and Annual Meeting theme each year. Once the overall theme and debate topics are selected, 2 student teams, each comprising a professional advisor and 3–5 students, are assigned a topic and stance. Members of the SDS are also assigned to each topic to provide an unbiased introduction. Over the course of 5–6 months, each team gathers evidence to defend their stance and prepare arguments in anticipation of the opposing team’s critiques. At the ESA Annual Meeting, the unbiased introduction and team stance arguments are presented, followed by a series of cross-examinations and rebuttals between the 2 teams. Finally, the audience and judges are given the floor to ask questions of the teams. New this year, teams were given the opportunity to also ask ‘interjected questions’ during the opposing teams’ rebuttal time, to encourage more lively and interactive discussion between the teams during the debate. Additionally, each team presented a final summary statement for their stance, and an SDS member provided an overall unbiased topic summary to conclude the topic debate. A panel of 3 judges, composed of ESA members, evaluated and scored the teams’ cohesiveness, use of time, and persuasiveness, as well as the quality of the teams’ presentations, prepared materials, questions, and answers.

This year, the SAC hosted a social media poll for ESA members to select their favorite debate topics. The topics were proposed by SDS members and ESA membership with the goal of aligning with the 2023 ESA Annual Meeting theme, ‘Insects and influence: Advancing entomology’s impact on people and policy’. Based upon popular vote, this year’s student debates theme was ‘Addressing emerging issues in entomology’, where 4 student teams debated the following topics:

(1) Should the disclosure of artificial intelligence (AI) large language models (LLMs) in scientific writing always be required?
(2) Is it more important to prioritize honey bee or native pollinator health for long-term food security within North America?

In the following article, we outline the 2023 ESA Student Debates by first providing each topics’ unbiased introduction from an SDS member, followed by the teams’ topic stance summaries.

## Should the Disclosure of AI LLMs in Scientific Writing Always Be Required?

### Unbiased Introduction by Jacqueline Maille and William Jacob Pitt

Scientific writing is the written communication of scientific content to peers and broader audiences (Heard 2022). Various types of scientific writing include research articles, scientific reviews, grant proposals, lab reports, white papers, extension documents, and conference proceedings.

In 2022, Open AI released the most advanced LLM to date, ChatGPT, making this technology publicly available to users around the world. An LLM is an advanced AI algorithm that is trained on enormous amounts of data and can generate sophisticated text responses based on user inputs (Thirunavukarasu et al. 2023). One of the many potential benefits of LLMs for entomological science, and science more broadly, is assistance with writing. Indeed, LLMs can generate large amounts of text and help scientists optimize language and grammar, draft paragraphs, summarize text, and refine manuscript structure (Castellanos-Gomez 2023, Ray 2023). This capability is rapidly improving with the advent of academic writing tools such as Sparks and Overleaf Copilot, which use LLMs to inspire creativity in an author’s writing (Gero et al. 2022, OverleafCopilot 2024). It is clear that LLMs have a high potential to assist scientists with academic writing.

However, there are ethical concerns surrounding LLM use. LLMs have inherent automation bias, in that users may favor ideas from LLMs simply because they are produced from an automated decision-making system (Jakesch et al. 2023). This bias has the potential to be overlooked, because authors may use these tools to reduce the fatiguing mental load in influencing, producing, editing, or reviewing scientific writing (Hosseini and Horbach 2023). Authors using inspired AI-written content may unknowingly be perpetuating biased stereotypes, cultural values or opinions echoed in the model from developers and user feedback features alike (Jakesch et al. 2023, Hofmann et al. 2024). Reporting LLM use could help identify where bias may be present.

However, LLMs can be used to help optimize language and grammar rather than generate ideas. Disclosure of LLM use in the preparation of a scientific manuscript could cause unwarranted reader skepticism about the work’s validity if the LLM use was not central to the design, results, or conclusions of the study. Therefore, the mandated disclosure of any LLM use could provide additional disadvantages to scientists who use these services simply for assistance with grammar. Additionally, other AI tools that assist with language and grammar have been in use for some time. For example, Grammarly (Grammarly Inc., San Francisco, CA), which was released in 2009 is an AI tool that detects writing flaws and suggests corrections for users to implement. Journal guidelines do not appear to provide proper disclosure of Grammarly use, so it is likely that this tool is frequently used without disclosure.

Concerns about the research ethics of LLMs have prompted many journals to develop guidelines for the proper use and disclosure of LLMs in scientific manuscripts. Many journals with guidelines on LLMs ban listing LLMs as a coauthor of a manuscript, as LLMs do not have the ability to be accountable for published research (COPE 2023, Hosseini et al. 2023, Rahimi and Talebi Bezmin Abadi 2023, Thorp 2023). Furthermore, some journals have specified that use of LLMs is required to be disclosed in a manuscript (Nature Portfolio 2023, PNAS Journals 2023), and some journals have banned the use of LLMs entirely (Science Journals 2023). The American Psychological Association (APA) writing style suggests disclosure of LLM use in the methods section of research articles (McAdoo 2023). Although some journals provide author guidelines on disclosure of LLM use, it is not clear if disclosure should always be required. This debate will address the question of whether the use of LLMs in the preparation of scientific writing should or should not be disclosed in every circumstance.

### Team 1 Stance: Yes, Disclosure of the Use of AI LLMs in Scientific Writing Should Always Be Required

Team Members: Tyler R.T. Musgrove, Ethan Doherty, Arjun Khadka, Allyson Martin Ewert, Tanner C. Sparks, Bandana Shrestha

Team Advisor: Dr. Blake Wilson, Louisiana State University

AI usage has become a debate across the sciences as LLM systems like ChatGPT develop and contribute to research and scientific communication (McVey 2022, Chrisinger 2023). While AI provides many potential benefits to its users, there are concerns regarding the ethics of utilizing AI in scientific writing due to the incorporation of false information, the potential for distrust in science, and the failure to properly cite work (Mittelstadt 2019, Ayling and Chapman 2022, Chen 2023, Elali and Rachid 2023, Frye and Chat GPT 2023, Hosseini et al. 2023, Salvagno et al. 2023). To add to these concerns, it is increasingly difficult for humans to discern between texts generated by AI and human writers (Gao et al. 2023). Thus, it is imperative to provide documentation of AI usage to mitigate adverse impacts.

The disclosure of AI usage provides greater transparency to the scientific field (Kabat 2017). As public distrust of science increases, not divulging AI usage could create a greater gap in scientific communication, while its proper documentation could create much-needed accountability and understanding surrounding the use of this new technology (Small and Mallon 2007, Kabat 2017, Desmond 2022). Not disclosing AI usage could unknowingly deceive or mislead both public and academic audiences with biases, poor quality or fabricated data, and plagiarism, among other issues (Kumar et al. 2014). Disclosing AI usage combats the potential for AI-generated errors to erode scientific credibility by clarifying the origins of potential inaccuracies that would otherwise be contributed to the scientists (Currie 2023). Similarly, the disclosure of AI usage in scientific writing clarifies authorship and intellectual ownership issues between human authors, since it allows for the proper identification of contributions to a paper (Teixeira Da Silva 2011). While the exact method of acknowledging AI contributions is disputed, it is clear that documentation of AI allows for personal accountability since it ensures that all readers and human authors are aware of the methods used for developing the paper, allowing for more ethical collaboration (Hosseini et al. 2023, Pourhoseingholi et al. 2023). We need to be transparent and descriptive in our usage of AI in order to establish both public trust and trust between scientists surrounding its usage.

Furthermore, adequate disclosure of AI use allows us to properly assess the value of scientific publications. While AI has the potential for increasing the efficiency of the scientific writing process through assisting with the writing style, formatting, and proofreading, other uses may involve utilizing AI in more fundamental aspects of scientific writing such as exploring the relevant literature, conducting statistical analyses, and developing relevant protocols (Fitria 2021, Thunström 2022, Culp 2023, Hosseini et al. 2023, Huang and Tan 2023, Tregoning 2023). In doing so, the dissonance between the skill levels and scientific understanding of researchers and the size of their work may become apparent (Salvagno et al. 2023). Essentially, unethical use of AI may allow researchers to become removed from the process of science while increasing perceived productivity and research experience (Frye and Chat GPT 2023, Salvagno et al. 2023, Sinclair 2023, Staiman 2023). With even experienced reviewers sometimes being unable to detect the use of AI for the falsification of abstracts, this may have long-reaching effects on the structure of academic promotion and tenure, which is largely based on topical expertise and experience (Dergaa et al. 2023).

Since AI is a relatively new tool within science, proper documentation allows for the tool to develop and grow with transparency (Satyam and Geetha 2023). This not only improves the efficiency and dependability of the tool but also allows scientists to collaborate and share resources and ideas (Golan et al. 2023, Salvagno et al. 2023). AI is widely used to powerfully cut down writing time by synthesizing information while highlighting overlooked data, so the issue lies in how we cooperatively use this tool to advance the field rather than opening the door for its abuse (Culp 2023, Dergaa et al. 2023, Hammad 2023, Stokel-Walker 2023). Thus, the disclosure of AI in scientific writing allows for greater innovation, transmission of ideas, and academic cooperation by allowing transparency and accountability as scientists in addition to clarifying intellectual ownership and publication value issues.

### Team 2 Stance: No, Disclosure of the Use of AI LLMs in Scientific Writing Should Not Always Be Required

Team Members: Kelly M. Tims, Carla-Cristina Edwards, Malcolm Peavy, Zia Valerie Williamson

Team Advisor: Dr. Kelly Carruthers, University of Georgia

The rapid evolution of AI LLMs, like ChatGPT, has given rise to ethical concerns surrounding the use of LLMs in writing. There is a growing debate on whether scientists should disclose the use of AI assistance. Here, we argue that utilizing LLMs in scientific writing does not always warrant disclosure.

LLMs have a wide spectrum of applications in writing, creating ambiguity about which applications warrant disclosure. Tools like Grammarly can be used to correct grammatical errors, improve readability, and provide text summaries (Perdana et al. 2021, Dang et al. 2022). Some LLM tools can translate text, aiding EFL researchers seeking to improve their publications in an English-dominated scientific community (Guo et al. 2022, Chen 2023, Ciaccio 2023, Zhao 2023). AI-assisted writing will likely become ubiquitous, especially as common word processors like Microsoft Word begin integrating AI assistance (Kelly 2023). Because generative LLMs are trained on massive text databases, there’s a concern that LLMs allow authors to plagiarize more freely (Nakazawa et al. 2022). However, Gao et al. (2023) found that ‘original’ (human-written) abstracts had higher plagiarism rates than AI-generated abstracts, with median plagiarism detector scores of 62.5% plagiarized vs 0% plagiarized, respectively. Regardless, disclosing whether a manuscript was written with AI does not exempt an author from anti-plagiarism regulations (Elsevier 2023, Nature Portfolio 2023).

Because AI-detection tools can be easily defeated with minimal effort (Gao et al. 2023), requiring authors to disclose AI usage would be unenforceable. Additionally, some AI-detection tools are more likely to wrongfully classify text written by non-native English speakers as AI-generated with a false positive rate around 61.8% (Liang et al. 2023). Enforcing the disclosure of every form of AI assistance would severely impede the publication process, which is already sufficiently arduous.

Requiring disclosure of any AI use in scientific writing may stigmatize authors using AI assistance. Among all scientific publications, 98% are in English (Ramírez-Castañeda 2020). Ramírez-Castañeda (2020) reported that 44% of EFL doctoral students have had their papers rejected from journals due to poor English. Therefore, LLM-based translation or English enhancement tools may prove especially useful for EFL scientists publishing in an English-dominated field. More concerningly, research has shown that a scientific study’s ‘perceived credibility’ can be unjustly altered based on how the research is framed to the public. A paper in the International Journal of Artificial Intelligence in Education demonstrated that when the same scientific evidence was presented within the framework of ‘AI’, it was viewed as less credible and less prestigious than when presented from a ‘neuroscience’ or ‘educational psychology’ framework (Cukurova et al. 2020). This indicates that authors forced to disclose AI use will be wrongfully discredited by negative biases, even when the research presented is sound and trustworthy (Cukurova et al. 2020). Disclosing LLM-assisted writing in any form may lead to unfair stigma against otherwise high-quality papers, and therefore could discourage researchers from taking advantage of this useful technology in the future. Stigmatization surrounding AI-assisted writing may perpetuate existing barriers against EFL scientists and may even discourage the use of these powerful productivity-enhancing tools wholesale.

LLM-based writing tools present an exciting opportunity for scientists to increase their productivity by reducing the intellectual rigor necessary in scientific writing. AI writing assistance can improve the quality of anyone’s writing, but especially those writing in a language foreign to them. Due to the ambiguity surrounding what level of AI writing assistance would require disclosure, the difficulty in enforcing disclosure of LLM usage, and the potential stigma against AI-written content unfairly affecting EFL writers, we argue that the use of LLMs in scientific writing should not always require disclosure.

## Topic 2: Is It More Important to Prioritize Honey Bee or Native Pollinator Health for Long-term Food Security Within North America?

### Unbiased Introduction by Jordan Twombly Ellis and Sara Salgado

Crop pollination is an extremely important economic service. It is estimated that, in the United States alone, animals provide 16 billion USD of pollination services. 12 billion USD of that is attributable to a single bee species, the European honey bee *Apis mellifera* (Khalifa et al. 2021). Insect pollination is necessary for the production of approximately 1,500 crops (Klein et al. 2007). It follows that agricultural yields and food security are heavily reliant on animal pollination (Ollerton et al. 2011). Legislatively and scientifically, much of the focus for pollinator protection is put on honey bees (Geldmann and González-Varo 2018).

Honey bees are non-native pollinators in North America and were introduced in the 17th century (Nielsen et al. 1994). Since their introduction, honey bees have become integral to North American crop pollination (Rucker et al. 2012). As pollinators, they have the unique attributes of perenniality, easy management, and transportation ability (Barrett et al. 2018). With each honey bee colony consisting of upwards of 10,000 bees, they provide an easy way to increase pollination services in a given area at a given time (Dornhaus et al. 2006). In 2023, US income attributed to pollination by honey bee hives was estimated at around 255 million USD (USDA 2024). Honey bees additionally are prolific honey producers and therefore the preferred species used by beekeepers in the United States, generating 377 million USD for honey producers in 2023 (USDA 2024). However, honey bees face stressors from their wide-scale use, such as pathogens, pesticides, and the parasitic mite, *Varroa destructor* (Oldroyd 2007, Yang and Cox-Foster 2007, Sanchez-Bayo and Goka 2016). The *Varroa* mite was introduced to North America in the 1980s, after which there was serious fear that honey bee populations and food security were in danger (Carreck et al. 2010).

Though *Apis mellifera* is the primary focus for pollination research, tens of thousands of pollinator species exist in North America. Native pollinators range from bees and wasps (Hymenoptera) to butterflies and moths (Lepidoptera), certain flies (Diptera), many beetles (Coleoptera), and even bats (Scrotifera), and hummingbirds (Apodiforms) (Requier et al. 2023). These pollinators have a long-evolved and intimate connection with the plants they pollinate (Mitchell et al. 2009). This often leads to certain pollinators being particularly suited to pollinate specific plants (Mateos-Fierro et al. 2022). Additionally, the range and diversity of native pollinators in North America offers a potential failsafe to crop pollination, as a singular challenge to one of these pollinators may not have the same detrimental impact on the others (Winfree et al. 2007).

Monocrop agriculture, where only 1 crop is grown each year for many acres, is becoming increasingly prevalent in North America (Jacques and Jacques 2012). The blooming period in monocrop operations happens for an isolated period of time (Cohen et al. 2021). For example, the almond bloom in California takes place over the course of a month from mid-February to mid-March (Connell et al. 2018). The almond groves in California cover 940,000 acres, maybe the sole floral resource in the area, and are only in bloom for a few weeks each year (Embry 2018). The paucity of floral resources for the majority of the year in these operations leads to a decline in native pollinator populations (Goulson et al. 2015). This decline in native pollinators leads to an increased need to bring in honey bee colonies. Therefore, in many large agricultural operations, there are few other pollinators for the temporally sparse floral resources aside from honey bees (Garibaldi et al. 2011).

The existence of honey bees and native pollinators is not necessarily mutually exclusive for pollination services. In fact, dual pollination can actually lead to higher yields (Koh et al. 2018). However, the prioritization of honey bees over native pollinators is likely to lead to major decline and even potential extinction in native pollinator populations (Morales et al. 2017). Whereas the prioritization of native pollinators would necessitate a large-scale and economically difficult change in agricultural practices (Kevan et al. 1990, Winfree et al. 2011). Here we present arguments for the benefits of prioritizing either honey bees or native pollinators in science and policy.

### Team 1 Stance: It Is More Important to Prioritize Honey Bee Health for Long-term Food Security Within North America

Team Members: Hazel Scribner, Navi Balthazor, Rachel L. Johnson, Chip Markwardt, Rupinder Singh

Team Advisor: Dr. Kristopher Silver, Kansas State University

Pollinators are an invaluable resource for food security, pollinating one-third of our global food supply at a value of 50 billion USD (Dias et al. 1999, Das et al. 2018, Hristov et al. 2020, Reilly et al. 2020). As US legislation crops up regarding protecting pollinators, the question is: which pollinators do we protect? Managed honey bees are not native to North America; however, they are well established and have beneficial traits that provide economic and agricultural value. Prioritizing managed hives is the most efficient and impactful strategy to achieve sustainable agricultural production.

Honey bees have a long history as an ideal pollinator for North American agriculture and offer several advantages in comparison to native pollinators. Honey bee-keeping in North America began in the 1600s and is well established (Calderone 2012, Barrett et al. 2018). Unlike colonial farms, the current agricultural landscape is based on monoculture where honey bees excel but which are less preferable for native pollinators (Carman and Jenkins 2016, Sáez et al. 2022). Honey bees can also be easily implemented in new areas to aid in pollination across diverse croplands (Greenleaf and Kremen 2006, Calderone 2012, Rucker et al. 2012). Additionally, domesticated honey bees are easier to transport, making them ideal for commercial use (Iwasaki and Hogendoorn 2021). Furthermore, native bees are a hundred-fold more expensive to manage in colonies (Calderone 2012).

To ensure food security, we need to bolster our food supply, and honey bees are the most valuable pollinators of our current crops. Of the 20 billion USD generated by insect-pollinated crops in the United States, almost two-thirds were pollinated by honey bees (Das et al. 2018). Most of the food we eat comes from crops grown in North America (FDA 2023). Many of these crops are directly dependent on bee pollination, while others are indirectly dependent (Calderone 2012). As global demand for insect-pollinated crops grows, pollinators, both native and non-native, are currently unable to keep up with this high demand (Aizen and Harder 2009). Evidence clearly shows that honey bees perform better in disturbed ecosystems such as managed cropland and non-native crops, which make up the majority of our agricultural system (Rollin et al. 2013, Carman and Jenkins 2016, Urbanowicz et al. 2020, Cruz et al. 2022, Sáez et al. 2022).

When the average person is told to name a pollinator, the first thing that comes to their minds is the honey bee (Barrett et al. 2018). Numerous campaigns about pollinators prominently featuring honey bees exist. The decline of honey bees in 2006 brought the health of honey bees to the forefront of public concern (Obama 2014, Barrett et al. 2018). Cass et al. (2022) suggest that public perception of pollinator sustainability lacks the distinction to differentiate between managed and native pollinators. Because of this, conservation efforts have focused primarily on honey bees and research on honey bee health has been heavily funded. Meanwhile, native pollinators are much less studied (Geldmann and González-Varo 2018). To shift to native pollinators now would require a massive overhaul, not only with the public perceptions of pollinators, but also in the field of pollinator research. Accordingly, it is prudent to allocate our limited resources towards more efficient management and utilization of honey bees for pollination. This approach ensures better pollination results and maximizes monetary benefits (Saunders et al. 2018). In addition, by wisely applying conservation efforts to honey bees, we simultaneously support the well-being of native pollinators (Halvorson et al. 2021).

Native pollinators are insufficient to pollinate the crops we already have and will not be enough as food production increases to meet our growing North American population. Honey bees are well established in agriculture, with an abundance of available research and past knowledge, and provide higher quality pollination services to many of the crops grown in North America. For successful conservation, public support is required, and honey bees are well-recognized as pollinators, which brings much-needed support and funding for sustainable policies. For conservation to be effective, we must focus funding on honey bees, rather than dividing it between many native pollinators.

### Team 2 Stance: It Is More Important to Prioritize Native Pollinator Health for Long-term Food Security Within North America

Team Members: Natalie Constancio, Kayleigh C. Hauri, John J. Ternest, Scott W. Gula, DeShae Dillard

Team Advisor: Dr. Zsofia Szendrei, Michigan State University

Pollinator-dependent crops provide necessary nutrients (e.g., vitamins, minerals, and lipids) that often cannot be obtained in sufficient quantities from other calorie-dense foods such as grains (Eilers et al. 2011, Katumo et al. 2022). However, the ability of these crops to meet consumer demand has diminished in recent decades, exacerbating food insecurity. In 2008, there was no shortage of pollination services in agriculture (Aizen et al. 2008) but increasing demand for pollinator-dependent crops combined with stagnant honey bee colony growth (Aizen and Harder 2009) has resulted in 5 of 7 investigated crops (sweet cherry, tart cherry, blueberry, apple, and watermelon) becoming pollinator-limited (Reilly et al. 2020). By definition, food security goes beyond meeting caloric needs: humans require access to sufficient, safe, and nutritious food meeting dietary needs and preferences while supporting a healthy lifestyle (Berry et al. 2015). The foundation of food security is availability, accessibility, utilization, and stability, which necessitate sustainability to meet present and future demand (Berry et al. 2015). To achieve long-term food security in North America, native pollinator health must be prioritized.

Native pollinators represent a diverse assemblage of species that provide reliable, resilient, and stable pollination services (Winfree et al. 2007, Blüthgen and Klein 2011). Conversely, honey bee visitation is variable, which can lead to inconsistent fruit set and yield (Brittain et al. 2013, Rader et al. 2016, Mallinger et al. 2021). Native pollinators are also more effective than honey bees in a variety of crops. In watermelon, when combining visitation rate and per-visit pollen deposition, native bees accounted for 62% of all pollen grain deposition and buffered against yield losses due to redundant services provided (Winfree et al. 2007). Unlike honey bees, many native pollinators buzz pollinate which is required for complete pollination of crops such as tomatoes, eggplants, and blueberries (Cooley and Vallejo-Marín 2021). Native pollinator communities also exhibit functional complementarity, where different species synergize to provide increased pollination services by occupying diverse ecological niches (Blüthgen and Klein 2011, Brittain et al. 2013). In the short term, crop yield increases when multiple pollinator species spatially segregate foraging preferences on different parts of the plant, or when various-sized bees pollinate different stigmas within a single plant (Blüthgen and Klein 2011). Long-term, functional complementarity in species-specific responses to disturbance can buffer pollination services against climate change (Blüthgen and Klein 2011). Pollination by a diverse range of native pollinators leads to higher fruit set and greater yield (Rader et al. 2016) which results in increased availability and stability of critical nutrient-rich foods.

To achieve food security, nutrient-rich foods must be accessed and utilized universally, but many communities exist within a food desert. For communities that lack access to nutritious foods, community gardens can supplement a healthy lifestyle by increasing daily fruit and vegetable consumption in both adults and children (Pawelek et al. 2009, Block et al. 2012, Carney et al. 2012). Prioritizing native pollinators in community gardens is a way to promote pollination services and native flower plantings and their associated management practices could serve to provide nesting habitat. By planting native flowers, 1 community garden experienced over a 4-fold increase in bee species over a single year (Pawelek et al. 2009). Investing in native pollinators ensures access to decentralized pollination services for all people without the costs of purchasing and managing honey bee hives. Relieving this financial burden allows communities within food deserts to access safe and nutritious food, enhancing food sovereignty.

The status quo of prioritizing honey bees falls short of addressing nutritional needs in North America. Food security is a global problem that permeates down to the individual, and native pollinators provide effective solutions across differing scales. Native pollinators increase yield while providing a buffer against climate change, improving the availability of nutritious food and stability of the food production system. Prioritizing native pollinators provides ecosystem services and complete crop pollination in community gardens, increasing accessibility and utilization for those who need it most. Native pollinators can meet the needs of the present without sacrificing the needs of future generations, thus providing a sustainable path towards long-term food security.

## Debates Summary

Day to day we are presented with new events and issues that continually change our lives. Entomology is encompassed within a global network of varying regions, industries, people, and ecosystems, challenging us to constantly contemplate the futures of the field. This year, the ESA chose to reflect on ‘Insects and influence: Advancing entomology’s impact on people and policy’ for the 2023 ESA Annual Meeting. This inspired the SDS and ESA membership to address emerging issues in entomology at the 2023 ESA Student Debates, in particular recent challenges facing the futures of scientific communication and food security.

The first half of this year’s student debates addressed the ethical issue of whether the disclosure of LLM use in scientific writing should always be required for scientific writing. Those in favor of disclosing LLM use in every circumstance argued that required disclosure is vital for greater transparency and accountability in science, which will prevent abuse and reduce negative public perceptions. Additionally, it was argued it could encourage scientific cooperation and innovation. On the other hand, those not in favor of disclosing LLM use in every circumstance argued that this requirement would create undue bias against the authors, discourage its use, is unenforceable, and would unfairly perpetuate existing barriers to publication by foreign language speakers.

In the second half, teams debated whether it is more important to prioritize honey bee or native pollinator health for long-term food security within North America. Those in favor of prioritizing honey bees emphasized the well-established history of honey bee use in North American agriculture, their enhanced crop performance, a more positive and popular public perception, and the concentration of limited resources on a thoroughly researched solution. In contrast, those in favor of prioritizing native pollinators for food security argued that native pollinators provide a diverse assemblage of species that increase yield and buffer against climate changes, decentralizes access to pollination services for growers at all production levels, and is a more sustainable food security solution for future generations while still meeting current demand.

Neither debate topic is particularly new to entomologists, and many members already have their own thoughts on these issues. Our hope is that the student debates fostered reflection and conversation among the entomological community on the emerging issues facing entomology and how it may impact people around the world, while additionally highlighting the exceptional students in our community who demonstrated skills in research, critical thinking, collaboration, and scientific communication while debating these topics. ESA and SAC are proud to provide this opportunity to students and believe the student debates are an excellent environment in which entomologists can continue to discuss topics of relevance to the future of entomology and the global community. Readers are encouraged to consider supporting future student debates at the ESA Annual Meeting through suggesting topics, attending the event, or even participating as team member or judge.
